# Quercetin ameliorates acute lung injury in a rat model of hepatopulmonary syndrome

**DOI:** 10.1186/s12906-022-03785-w

**Published:** 2022-12-03

**Authors:** Noha Abdel-Aziz Nassef, Manal S. Abd-El Hamid, Samy A. Abusikkien, Asmaa Ibrahim Ahmed

**Affiliations:** 1 Assistant Professor of Physiology, Faculty of Medicine, Ain Shams University, Cairo, Egypt; 2 Lecturer of Anatomy, Rabigh Faculty of Medicine, King Abdulaziz University, Faculty of Medicine, Ain Shams University, Cairo, Egypt; 3 Assistant Professor of Anatomy, Faculty of Medicine, Ain Shams University, Cairo, Egypt

**Keywords:** Quercetin- hepatopulmonary-endothelin B receptor-bile duct ligation

## Abstract

**Background:**

Common bile duct ligation (BDL) is a rat experimental model to induce biliary cirrhosis. Lung fibrosis and pulmonary vascular angiogenesis and congestion are the most common complications of biliary cirrhosis that is known as hepatopulmonary syndrome. The aim of the present work is to investigate the acute lung injury in a BDL model and to investigate the possible protective effect of quercetin on this injury.

**Methods:**

Twenty-four adult male albino rats of the Wister strain (weighing 150–250 g). Animals were divided into 3 groups, with 8 rats each: Group I: Sham-operated group (control). Group II: Bile duct ligation group (BDL) sacrificed after 28 days from the surgery. Group III: Quercetin-treated bile duct ligation group (Q-BDL) was given orally by gastric gavage in a dose of 50 mg/kg/day, starting from the 4th day of the operation until the 28th day. At the end of the experiment, at day 28, all rats were sacrificed. Lung specimens were processed to measure Endothelin B receptor gene expression by PCR, lung surfactant by ELISA, “eNO” s by immunohistochemistry. Histological assessment was done using; H&E, Masson’s trichrome, PAS, toluidine blue-stained semi-thin sections, transmission electron microscope. Histomorphometric and statistical studies were done.

**Results:**

BDL group showed significant increase in lung index together with mononuclear cellular infiltration denoting lung inflammatory state. Also, the significant increase in pulmonary endothelial nitric oxide synthase ("eNO" s) area percent and endothelin B receptor (ET_B_) gene expression indicates enhanced angiogenesis. Pulmonary surfactant concentration was significantly decreased together with thickening of interalveolar septa denoting lung injury and fibrosis. Quercetin led to significant decrease in lung index, pulmonary "eNO" s area percent, ET_B_ gene expression and significant increase in pulmonary surfactant concentration. Quercetin treatment improved histological changes and morphometric measurements, limited mononuclear cellular infiltration and decreased perivascular and perialveolar collagen deposition.

**Conclusion:**

Quercetin ameliorates the hepatopulmonary syndrome-induced lung injury through its anti-inflammatory, antioxidative and antifibrotic effects.

## Background

Hepatopulmonary syndrome (HPS) is a serious pulmonary vascular complication of cirrhosis that markedly increases morbidity and mortality [1]. Much of our knowledge arises from studies on rat experimental models in which common bile duct ligation (BDL) has been performed in order to develop secondary biliary cirrhosis [2].

The primary feature of HPS is the intrapulmonary vasodilatation and shunting that result in hypoxemia and worsen the quality of life [3]. These changes may be induced by increased nitric oxide (NO) production synthesized by endothelial nitric oxide synthase ("eNO" s) or by inducible nitric oxide synthase ("iNO" S) produced by the infiltrating monocytes [2]. It was found that NO inhibition with N-nitroarginine methyl ester (L-NAME) decreases intrapulmonary shunting and improves gas exchange [4].

Endothelin-1 (ET-1) plays a significant role in the pathophysiology of HPS. Although ET-1 is a potent vasoconstrictor, it may promote vasodilation in the pulmonary circulation of BDL rats. The differential effect of ET-1 depends on the expression of and binding to its receptors. The endothelin A (ET_A_) or B (ET_B_) receptors expressed by vascular smooth muscle cells mediate vasoconstriction, while the ET_B_ receptor on endothelial cells upregulates "eNO" s and NO production and mediate vasodilation [5]. In BDL rats, ET_B_ receptors are specifically upregulated, probably driven by increased pulmonary shear stress during the hyper-dynamic condition of liver disease [6]. Further, endothelin might increase nuclear factor-kB levels enhancing the expression of adhesion molecules and inflammatory cytokines [7]. Inflammatory cells such as monocytes may activate the vascular endothelial growth factor A (VEGF-A) which enhance angiogenesis that plays a role in the pathophysiology of HPS [8]. Increased plasma endotoxins, pro-inflammatory and oxidative markers in BDL might be implicated in pulmonary vascular dilatation and angiogenesis [6].

Decreased alveolar ventilation was also found to complicate the disease state. In a rat model of experimental HPS, alveolar type II cells showed apoptosis and decrease in surfactant production [9].

Despite significant progress in HPS research, liver transplantation is the only curative treatment, highlighting the need for novel effective medical therapies. Quercetin, a flavonoid that is abundant in human diet is known for its antioxidant and anti-inflammatory properties [7]. A previous clinical trial with COPD subjects showed that quercetin consumption was safe and well tolerated by the subjects [10]. Flavonoid acts to prevent lipid peroxidation by neutralizing various reactive oxygen species and by activation of the antioxidant enzymes thus it may prevent redox imbalance and lung inflammation [11].

However, its effect on pulmonary surfactant and alveolar type II cells in hepatopulmonary syndrome was not investigated.

Further, Quercetin was also found to inhibit angiogenesis in a rat model of common bile duct ligation [12]. Quercetin was able to inhibit macrophage activation and attenuate liver inflammation and fibrosis in a carbon tetrachloride mouse model [13]. The Quercetin ability to limit liver cirrhotic evolution might decrease the severity of the subsequent HPS [7]. However, this protective mechanism of quercetin is still doubtful in the lung tissue. Recently Araújo et al. [14] suggested that quercetin protected alveolar structure and halted inflammation and oxidative stress in a chronic obstructive pulmonary disease induced by cigarette smoke.

Emerging from the above points, the current work was aimed to investigate the effect and mechanism of action of quercetin administration, on lung function and morphology in experimentally induced hepatopulmonary syndrome, which has been performed by bile duct ligation to develop secondary biliary cirrhosis.

## Methods

### Animals

The research protocol and all experimental procedures comply with the guidelines of the research Ethical Committee of Faculty of Medicine, Ain-Shams University and with the revised Animals (Scientific Procedures) Act 1986 in the UK and Directive 2010/63/EU in Europe [15, 16]. Twenty-four adult male albino rats (weighing 150–250 g) were purchased from the experimental animal farm (Giza, Egypt) and housed in the Medical Ain-Shams Research Institute (MASRI), with suitable ventilation, temperature of 22–25 °C, 12 hours light dark cycle and free access to food and water. The animals were acclimatized to the new environment for one week prior to experimental procedures. Rats were classified randomly into the following groups (8 rats each):

1. Group I: Sham-operated group (Sham): rats underwent all surgical procedures without bile duct ligation, 28 days before sacrifice. Rats were given distilled water daily by gastric gavage, starting from the 4th day of the operation until the 28th day.
2. Group II: Bile duct ligation group (BDL): Bile duct ligation 28 days before sacrifice. Rats were given distilled water daily by gastric gavage, starting from the 4th day of the operation until the 28th day.
3. Group III: Bile duct ligation + Quercetin group (Q-BDL): Quercetin (3, 3′, 4′, 5, 6-Pentahydroxyflavon, Sigma, St. Louis, Missouri, USA) was prepared by freshly dissolving the daily dose in distilled water and then was given orally by gastric gavage in a dose of 50 mg/kg/day, starting from the 4th day of the bile duct ligation operation till the 28th day [17]. Rats in groups I and II were given the same volume of distilled water daily.

### Bile duct ligation and induction of obstructive jaundice

Animals were anesthetized with intraperitoneal ketamine (0.1 mg/g) and xylazine (0.01 mg/g). A midline abdominal incision was made, and the common bile duct was identified. The duct was dissected carefully by the naked eye and a single ligation of the bile duct just before its entry to the intestine was made, and the abdominal incision was closed in two layers [18]. In the sham-operated group, the duct was dissected without ligation, and all other steps were performed as in BDL group.

### Experimental procedures

On the day of sacrifice, overnight fasted rats, except for free access to water, were weighed and anaesthetized with intraperitoneal ketamine (0.1 mg/g) and xylazine (0.01 mg/g). Lungs were carefully dissected of excess surrounding tissue and weighed to calculate lung indices. Lung index = lung weight (mg)/body weight (g) according to Zhao [19], it was done to examine for the presence of pulmonary inflammation, congestion or edema. Hearts were also excised, weighed, and cardiac indices were calculated by dividing the absolute heart weight (mg) by the final body weight (g) to exclude cardiac causes of pulmonary congestion. Lung specimens were taken and divided; some specimens were fixed for paraffin embedding, and some specimens were stored at − 20°C for biochemical tissue assay of endothelin B (ETB) receptor expression and surfactant concentration.

### Quantitative real-time PCR for determination of endothelin B (ET_B_) receptor gene expression similar to Balyakina et al., [20]

Tissue samples of the studied groups were lysed and total RNA was isolated with RNeasy purification reagent (Qiagen, Valencia, CA). The purity of total RNA was measured with a spectrophotometer and the wavelength absorption ratio (260/280 nm) was between 1.8 and 2.0 for all preparations. Reverse transcription of total RNA to cDNA was carried out with a reverse transcription reaction (Superscript II, Gibco Life Technologies, Grand Island, NY, USA). Real-time PCR amplification and analysis were carried out using an Applied Biosystem with software version 3.1 (Step One, TM, USA). The reaction contained SYBR Green Master Mix (Applied Biosystems). The data were analyzed with the comparative cycle threshold (CT) method. The expression of β-actin mRNA was used as an internal control in all samples. The primers used were shown in Table 1.

**Table 1:** Primer sequences of studied genes

Gene	Sequence
Endothelin B receptor	Forward primer: 5′ -GATACGACAACTTCCGCTCCA- 3′ Reverse primer:5′ -GTCCACGATGAGGACAATGAG - 3’
Beta actin	Forward primer: 5’ - GACGGCCAGGTCATCACTAT − 3′ Reverse primer: 5′ - CTTCTGCATCCTGTCAGCAA − 3’

Determination of pulmonary surfactant in lung tissue was performed using Rat Pulmonary Surfactant-Associated Protein D (SP-D) ELISA Kit, supplied by MyBioSource, USA, according to the method provided by the manufacturer.

## Histological study of lung

### Light microscopic study Hematoxylin & Eosin (H&E), Masson’s trichrome (MTC), and periodic acid shift (PAS stain)

Lung specimens were fixed in 10% buffered formalin (pH 7.4) fixative solution for 24 hours, dehydrated in ascending grades of ethanol, cleared in xylol, and embedded in paraffin. Tissues were processed for preparation of paraffin blocks to get paraffin sections (5 μm in thickness), which were stained by conventional H&E to assess the cell nucleus, cytoplasm and organelles [21], Masson’s Trichrome that allows the differentiation of collagen fibers [22] and periodic acid shift (PAS stain) to assess glycogen presence which reflects the state of energy stores [23, 24].

### Light microscopic study, preparation of specimens for semi-thin sections and toluidine blue staining

The remaining part of lung tissue was immediately cut into cubes (1 mm in diameter) and fixed overnight in 2.5% phosphate-buffered glutaraldehyde (pH 7.3) at 4 °C. Post-fixation in 1% buffered osmium tetroxide for 1–2 h was followed by dehydration in ascending grades of ethyl alcohol, cleared in propylene oxide, and finally embedded in fresh Epon capsules. Semi-thin sections 1 μm in thickness were cut with a glass knife and stained with toluidine blue, and then examined by an Olympus light microscope. These sections were done to highlight presence of inflammatory cells [25, 26].

### Transmission electron microscopic (TEM) preparation of specimens for ultra-thin sections

Cubes (1 mm in diameter) of lung tissue were fixed in 0.1 M sodium cacodylate buffer containing 2.5% glutaraldehyde and 2% formaldehyde. After 2 h of fixation at 4 °C with 2% osmium tetroxide in 50 mM sodium cacodylate (pH 7.2), the specimens were stained overnight with 0.5% aqueous uranyl acetate. Specimens were dehydrated, embedded in Epon 812, and sectioned into ultrathin slices with ultra-microtome. The ultrathin sections were evaluated on a Zeiss Transmission Electron Microscope EM 900 [27].

### Determination of "eNO" s by immunohistochemistry staining of lung tissue

Five-micrometer-thick paraffin sections were mounted on positively charged slides and subjected to the immunohistochemical procedure using an Avidin-Biotin detection system (Ventana, Tucson, AZ, USA), following the manufacturer’s instructions. Sections were incubated with polyclonal guinea pig anti-"eNO" s antibody (1:100) (N1542, Dako, Carpinteria, CA, USA) performed by an automatic immunostainer (Ventana Bench Mark XT, Ventana) [28, 29].

### Computerized quantitative morphometric analysis (image analysis)

1. The thickness of interalveolar septa and pulmonary vessels (indicated by the distance diameter) performed in the H&E stained sections
2. In Masson’s trichrome stained sections, the area percent of collagen fibers was measured using binary mode
3. The number of pneumocyte type II was counted in the toluidine blue stained semithin sections
4. Area percentage of "eNO" s relative immunostaining density was done in "eNO" s immunostained sections

Morphometric measures were carried out using the Image Pro plus image analyzer computer system Leica DM2500 microscope with a built-in camera (Wetzlar, Germany). All images were digitally acquired using an image analyzer Leica Q win V.3 program (Wetzlar, Germany) installed on a computer in the Histology Department, Faculty of Medicine, and Ain Shams University. Five different nonoverlapping fields from five different stained sections were examined in each group for measuring each parameter.

Morphometric parameters that were measured at a high-power field of magnification are as follows:

Thickness of the inter-alveolar septa (μm).

Thickness of the wall of pulmonary vessels.

The area percentage of collagen fibers.

Area % of "eNO"s relative immunostaining density.

The mean number of type II pneumocytes/HPF.

### Semiquantitative lung injury score was done to reflect the severity of lung damage according to Silva et al. [30]

#### Statistical analysis

Statistical analysis was performed using SPSS statistical software, version 15.0 (SPSS Inc., Chicago, IL, USA) for Windows. Data were analyzed and presented as means± SD. Differences between continuous data were analyzed using one-way ANOVA. Correlation and lines of regression were calculated by linear regression analysis, Pearson correlation coefficient was calculated, and *P* < 0.05 was considered significant [31].

## Results

### Lung and cardiac indices

#### Lung weight

**Fig. 1:**
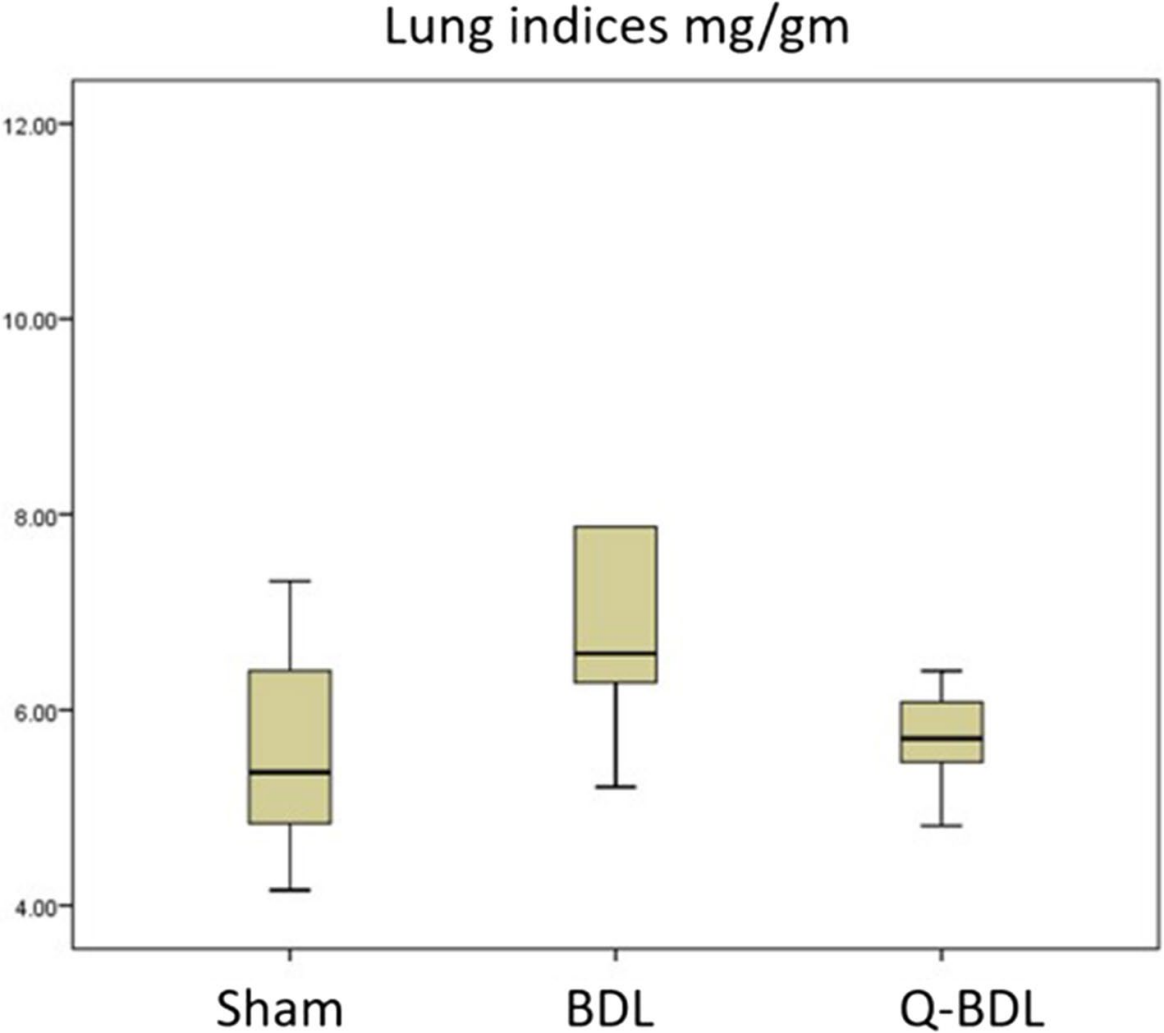
Box plot demonstrating lung indices in sham-operated group (sham), Bile duct ligation group (BDL) and Bile duct ligation + Quercetin group (Q-BDL)

In the present work, there was a significant increase in the lung weight normalized to body weight (lung index) in the BDL group compared to the sham group, which decreased back to the control values in the quercetin treated group (*P* < 0.05). The results of cardiac index showed that there was no statistical difference between BDL group compared to the sham group (Table 2, Fig. 1).

**Table 2:** mean ± standard deviation values of lung and heart indices (mg/g) in lung tissue of sham, bile duct ligation (BDL) and quercetin-treated bile duct ligation (Q-BDL) groups

	Sham	BDL	Q-BDL
Lung indices	5.6 ± 1.15	7.3 ± 2.18^a^	5.7 ± 0.52^b^
Heart indices	3.52 ± 0.51	3.66 ± 0.55	3.28 ± 0.44

a: Significant at *P* < 0.05 when compared to sham-operated group calculated by ANOVA, least significant difference. b: Significant at P < 0.05 when compared to BDL group calculated by ANOVA, least significant difference

### Biochemical analysis

**Fig. 2:**
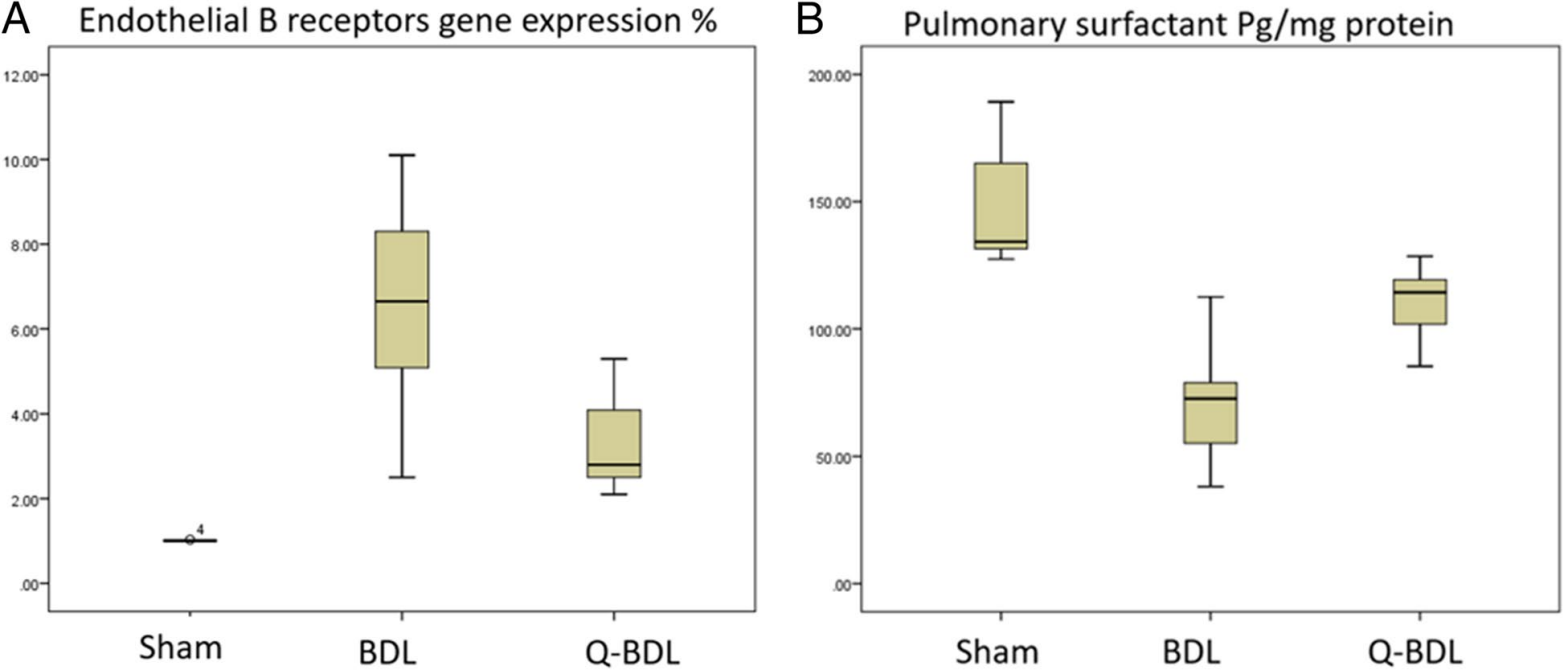
Box plot demonstrating a) Endothelin B receptor b) surfactant concentration in sham-operated group (sham), Bile duct ligation group (BDL) and Bile duct ligation + Quercetin group (Q-BDL)

Endothelin B receptor gene expression was significantly increased in BDL group compared to the sham group (*P* < 0.001). However, in the quercetin treated group this was significantly reduced compared to the untreated group (*P* < 0.005) (Table 3, Fig. 2).

**Table 3:** mean ± standard deviation values of endothelin B receptors gene expression (%) and pulmonary surfactant (pg/mg protein) in lung tissue of sham, bile duct ligation (BDL) and quercetin-treated bile duct ligation (Q-BDL) groups

	Sham	BDL	Q-BDL
Endothelin B receptors	1 ± 0.012	6.5 ± 2.464^a^	3.43 ± 1.193^b^
Pulmonary surfactant	146.9 ± 24.7	73.4 ± 22.3^a^	109.1 ± 15.2^b^

a: Significant at *P* < 0.05 when compared to sham-operated group calculated by ANOVA, least significant difference. b: Significant at P < 0.05 when compared to BDL group calculated by ANOVA, least significant difference

Pulmonary surfactant concentration in lung tissues was significantly reduced in BDL group compared to the sham group (*P* < 0.001). However, in the quercetin treated group this was significantly increased compared to the untreated group (P < 0.005) (Table 3, Fig. 2).

**Fig. 3:**
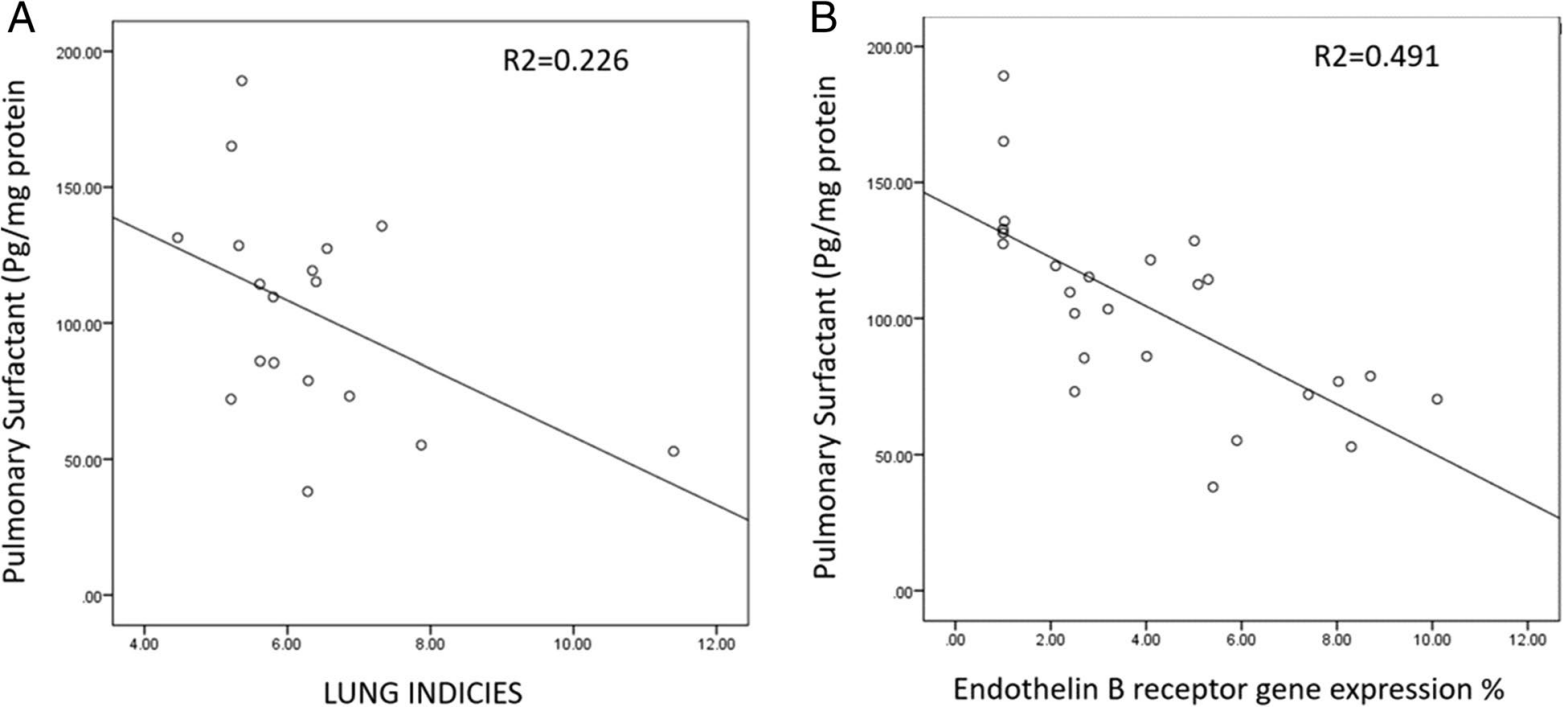
Regression lines and coefficients of determination (R^2^): It shows significant negative correlation between surfactant concentration in lung tissues and each of lung index and gene expression of endothelin 1-B receptors

There was a significant negative correlation between lung index and surfactant concentration in lung tissues (r = − 0.476, *P* < 0.05) in the data pooled from the three studied groups (Fig. 3). There was a significant negative correlation between endothelin B receptor gene expression and surfactant concentration in lung tissues (r = − 0.701, P < 0.001) in the data pooled from the three studied groups (Fig. 3).

### Histological results

#### The sham-operated group

**Fig. 4:**
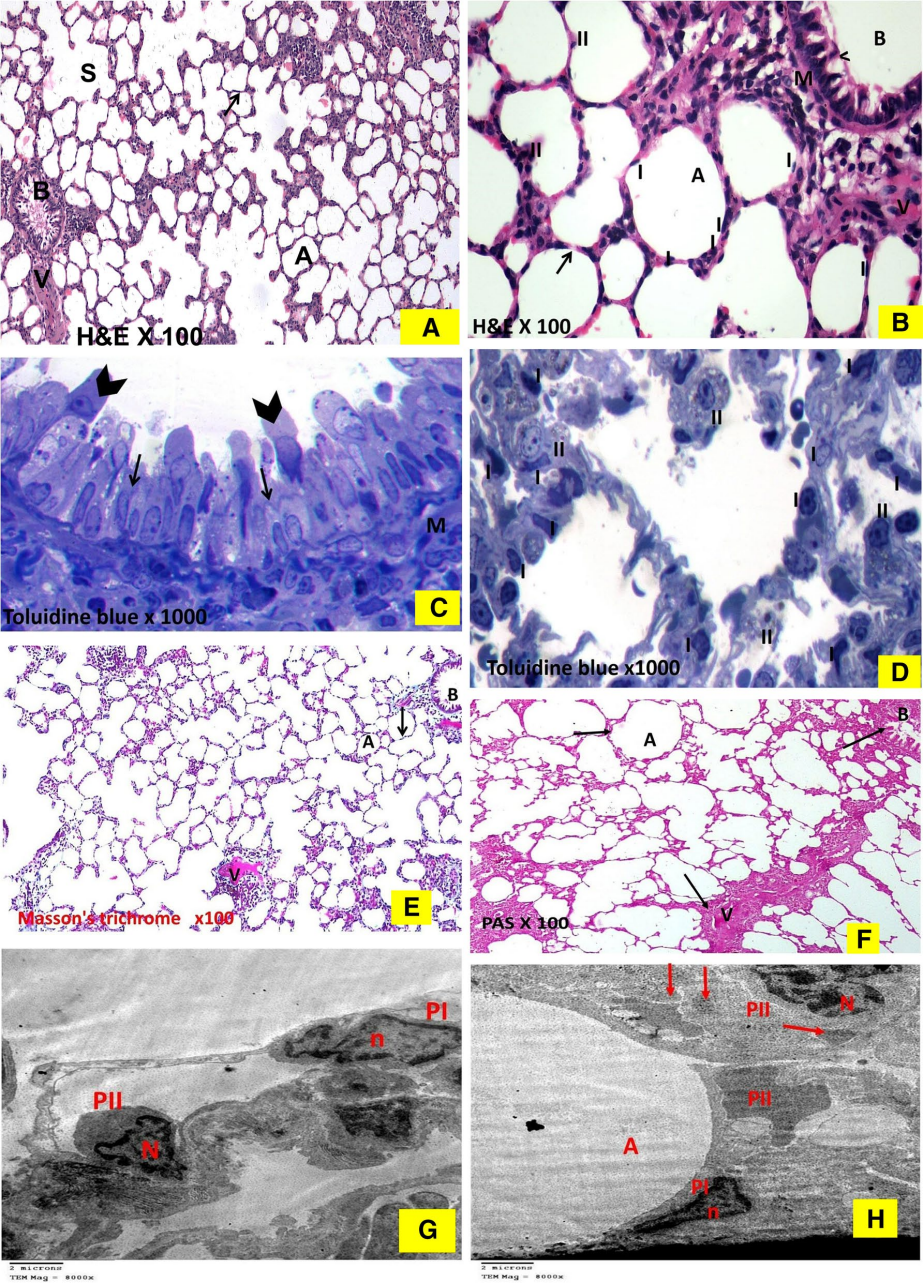
Histological changes in sham-operated group.The hematoxylin and eosin-stained sections. 4A. **Sham group** shows normal lung architecture with thin interalveolar septa (↑). Notice patent alveolar sacs (S), alveoli (A), bronchiole, (B) and pulmonary vessels (V). (× 100). 4B. **Sham group** shows thin interalveolar septa (↑). The bronchiole (B) is lined by simple columnar ciliated epithelium (*^*) and is surrounded by concentric layers of smooth muscle fibers (M). The alveoli (A) are mostly lined by thin type I pneumocyte with their flat nuclei (I) and few cubical pneumocytes type II with their large, rounded nuclei and vacuolated cytoplasm (II) present at the angles of interalveolar septa and pulmonary vessels (V). (× 400). Toluidine Blue stained semi thin sections: 4C. **Sham group** shows a terminal bronchiole, its epithelial lining showed two types of cells; ciliated cells (arrow) and non-ciliated cells (Clara cells) (arrowhead), with dome shaped apex protruding into the lumen and has narrow base. It had more darkly stained oval indented nucleus, slips of smooth muscle (M) are seen surrounded the bronchiole also can be seen. (× 1000). 4D. **Sham group** shows interalveolar septa containing capillaries and few inflammatory cells separating wide air spaces. The alveoli seen lined by mostly with flat pneumocyte type I (I) and few cubical pneumocytes type II with their large, rounded nuclei and vacuolated cytoplasm (II) present at the angles of interalveolar septa. (× 1000). 4E. Masson’s trichrome stained lung sections: **Sham** group has few green collagen fibers (↑) surrounding mainly the walls of the blood vessels (V). Notice scanty collagen fibers present in the alveolar septa (↑) and around the bronchiole (B). A = alveoli. (x100) 4F. The sham-operated group shows strong positive PAS reaction. In Photomicrographs of Ultrastructure examination by Transmission electron microscope (TEM) of lung: G. Transmission electron micrographs of the sham group showing patent alveolus (A) showing type-I of pneumocyte type-I (PI) with thin attenuated cytoplasm toward the alveolar space and flattened nucleus (n). dome shaped type-II pneumocyte (PII) with large euchromatic nucleus (N) and many lamellar bodies (blue arrow). (Uranyl acetate and Lead citrate × 8000). 4H. showing type-I of pneumocytetype-I (PI) with thin attenuated cytoplasm toward the alveolar space and flattened nucleus (n). Dome shaped type-II pneumocyte (PII) with large euchromatic nucleus (N). (Uranyl acetate and Lead Citrate × 8000)

As shown in (Fig. 4), the H & E-stained sections showed the normal spongy architecture of the lung. This appeared in the form of bronchi, bronchioles, alveoli and alveolar sacs separated by thin inter-alveolar septa (Fig. 4A). The terminal bronchioles were lined by simple columnar ciliated epithelium and surrounded by concentric layers of smooth muscle fibers. Majority of the alveoli were lined by thin type I pneumocytes with their flat nuclei while few alveoli were lined by cubical pneumocytes type II with their large, rounded nuclei that were located at the angles of the interalveolar septa (Fig. 4B).

1. In the Toluidine blue-stained semi-thin sections, epithelial lining of the terminal bronchiole showed two types of cells. Ciliated cells and non-ciliated cells (Clara cells). Clara cells appeared with dome shaped apex protruding into the lumen and has narrow base. It had darkly stained oval indented nucleus. Slips of smooth muscle cells surrounding the bronchioles was also seen (Fig. 4C). The *alveolar epithelium* was formed of both type I with attenuated cytoplasm and type II pneumocytes with vacuolated cytoplasm & projecting above the level of the surrounding epithelium (Fig. 4D).
2. Masson’s trichrome stained sections showed very thin collagen fibers in the interalveolar septa and surrounding the blood vessels & bronchioles. (Fig. 4E). Strong positive PAS reaction was detected in the bronchiolar passages, wall of a blood vessel, and walls of alveoli in the periodic acid- shiff (PAS) stained sections (Fig. 4F). In transmission electron microscope patent alveolus showed type-I pneumocyte with its thin attenuated cytoplasm toward the alveolar space and flattened nucleus, together with dome shaped type-II pneumocyte with its large euchromatic nucleus and many full lamellar bodies was also noted in a form of electron dense granules& normal microvilli (Fig. 4G) & (Fig. 4H).

#### Bile duct ligation group

**Fig. 5:**
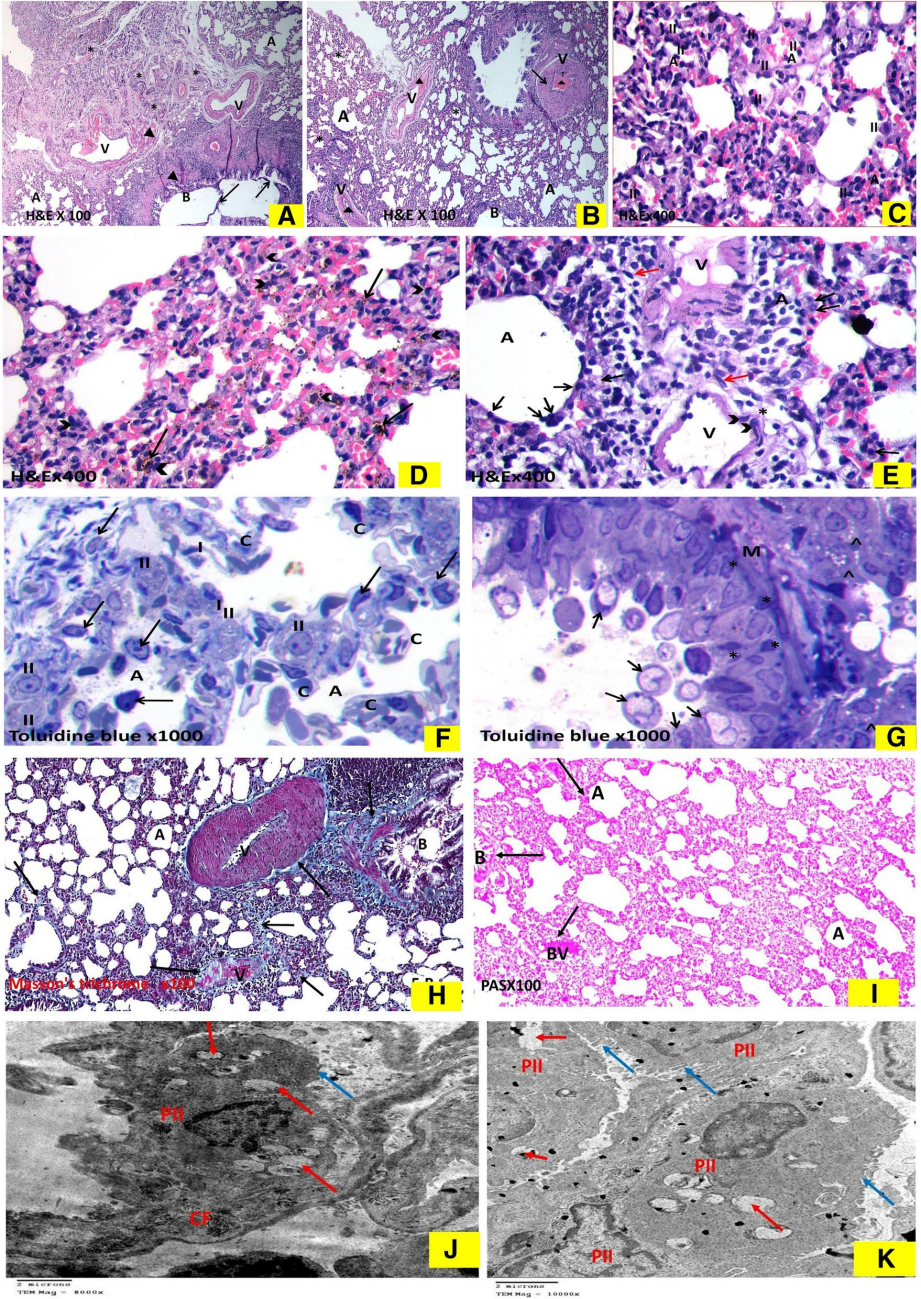
Histological changes in BDL group: The hematoxylin and eosin-stained sections: 5A. BDL group shows diffuse lung tissue affection, the hallmark is the widespread angiogenesis (*) in the form of multiple small-dilated congested blood vessels (V) variable in size and shape. The detached bronchiolar epithelium (↑) of the disfigured terminal bronchioles (B) together with peri bronchial and peri vascular smooth muscle hypertrophy (▲) observed. (A) = emphysematous dilated alveoli(× 100). 5B. BDL group shows some of the pulmonary blood vessels (V) showing thickened media (↑) and narrow lumen congested with bluish thrombus (T) and inflammatory infiltrate & exudate that appear as acidophilic structure less homogenous material (▲). Note the honeycomb appearance of the lung as some of the alveoli are narrow (*), while others show emphysematous dilatation (A), disfigured terminal bronchioles = (B) (× 100). 5C. BDL group shows thickened inter alveolar septa (*) that are infiltrated by RBCs & heavy mononuclear cellular infiltration. Apparent increase in the number of pneumocyte II (II) in distorted alveoli (A) are detected(× 400). 5D. BDL group  shows the presence of extravasated RBCs in the alveolar spaces, many macrophages (*^*) with their large eccentric nuclei and acidophilic cytoplasm laden with brownish hemosiderin granules (↑) are also infiltrating the thickened inter alveolar septa. (× 400). 5E. BDL group shows the air spaces (A) lined with large cell most probably pneumocyte type II (↑). Note the disruption (*) of the endothelial cell lining of the intima of some pulmonary blood vessel (V) with polymorphic mononuclear inflammatory cells (˄) adherent to it. Note the thickened inter alveolar septa by proliferating fibroblast (red arrow). (× 400). Toluidine Blue stained semi thin sections: 5F. A photomicrograph of a semi thin section of a rat lung of BDL group showing thick interalveolar septa separating air spaces (A) containing capillaries (C) and inflammatory cells (↑). The alveoli mostly lined by cubical pneumocytes type II with their large, rounded nuclei and numerus tiny vacuoles in its cytoplasm (II). They are present at the angles of interalveolar septa and hardly detected degenerated flat pneumocyte type I (I). (× 1000). 5G. BDL group showing a bronchiole lined by disfigured epithelial cells with deeply stained nuclei (*). The bronchiolar lumen is full of exfoliated epithelial cells and pale stained active macrophage cells (↑). M = smooth muscle, (˄) = interstitial foamy macrophage. (× 1000). Masson’s trichrome stained lung sections: 5H. The BDL group reveals apparent increase in the thick collagen bundles deposition (↑) in the lung interstitium including mainly the walls of the blood vessels (V) that have a narrow-congested Lumina and thickened media with smooth muscle hypertrophy. Moreover, thickened interalveolar septa (↑) and the lamina propria of the bronchioles (B) by apparent increase in the collagen fibers deposition are noticed (denoting lung fibrosis) as compared to that of sham group, alveoli = (A). (x100) 5I. The BDL group reveals week PAS reaction (x100). Photomicrographs of Ultrastructure examination by Transmission electron microscope (TEM) of lung: In BDL group. 5J. BDL group showing numerus variable sized depleted empty electron dense lamellar bodies (red arrow) in some of disfigured dome shaped type-II pneumocyte (PII) with peripheral clumps of heterochromatin of the nucleus i.e. inactive nucleus. Note also ill-defined blunt flattened microvilli of type-II pneumocyte (blue arrow) and nearby collagen fibrils (CF). (Uranyl acetate and Lead Citrate × 8000). 5K. BDL group reveals other type-II pneumocyte (II) showing signs of proliferation in the form of apparent increase in number, increase in microvilli (blue arrow) with numerus empty lamellar bodies (red arrow). (Uranyl acetate and Lead Citrate × 10000)

As shown in (Fig. 5), the H & E-stained sections revealed diffuse lung tissue affection. The hallmark was the widespread vascular congestion, angiogenesis in the form of multiple small-dilated congested blood vessels variable in size and shape. Detached bronchiolar epithelium of the disfigured terminal bronchioles together with mural smooth muscle hypertrophy observed (Fig. 5A). Moreover, some of the pulmonary blood vessels that had thickened media and narrow lumen filled with bluish thrombus and inflammatory exudate that appeared as acidophilic structureless homogenous material. The lung demonstrated honeycomb appearance as some of the alveoli were narrow, while others were dilated showed compensatory emphysematous dilatations (Fig. 5B). The inter alveolar septa were thickened and infiltrated by red blood cells (RBCs) and heavy mononuclear cellular infiltration, with apparent detected increase in the number of pneumocytes II in the distorted alveoli (Fig. 5C). Alveolar spaces had extravasated RBCs. Many macrophages with large eccentric nuclei and acidophilic cytoplasm laden with brownish hemosiderin were observed infiltrating the thickened inter-alveolar septa (Fig. 5D). Figure 5E revealed disruption of the endothelial cell lining of the intima of some pulmonary blood vessels with polymorphic mononuclear inflammatory cells adherent to it was noted. Inter-alveolar septa thickened by proliferating fibroblast.

Examination of the toluidine blue stained semi-thin sections of the BDL group revealed generalized thickening of the inter-alveolar septa with capillaries and inflammatory cells infiltrates. The alveoli are mostly lined by pneumocytes type II with hardly detected flat pneumocytes type I (Fig. 5F). The epithelial lining of the terminal bronchioles had sometimes darkly stained nuclei. Most of bronchiolar lumina appeared full of exfoliated epithelial cells and pale stained active macrophage cells (Fig. 5G). Masson’s trichrome stained sections showed apparent increase in the thick collagen fibers deposition in the lung interstitium including mainly walls of the blood vessels, alveoli, interalveolar septa, and lamina propria of the bronchioles (Fig. 5H). Periodic acid- shiff (PAS) stained sections revealed weak PAS reaction in the bronchiolar passages, wall of a blood vessel, and walls of alveoli (Fig. 5I). Transmission electron microscope of the BDL group revealed numerus variable sized depleted empty electron lucent lamellar bodies seen in some of disfigured dome shaped type-II pneumocyte with (peripheral clumps of heterochromatin of the nucleus) i.e., inactive nucleus. There were dilated cisternae of rough endoplasmic reticulum also ill-defined blunt flattened microvilli of type-II pneumocyte (Fig. 5J). Also, Type-II pneumocyte had signs of nonfunctioning proliferation in the form of apparent increase in their number, but, with numerus electron lucent empty lamellar bodies & microvilli (Fig. 5K).

#### The quercetin-treated bile duct ligation group

**Fig. 6:**
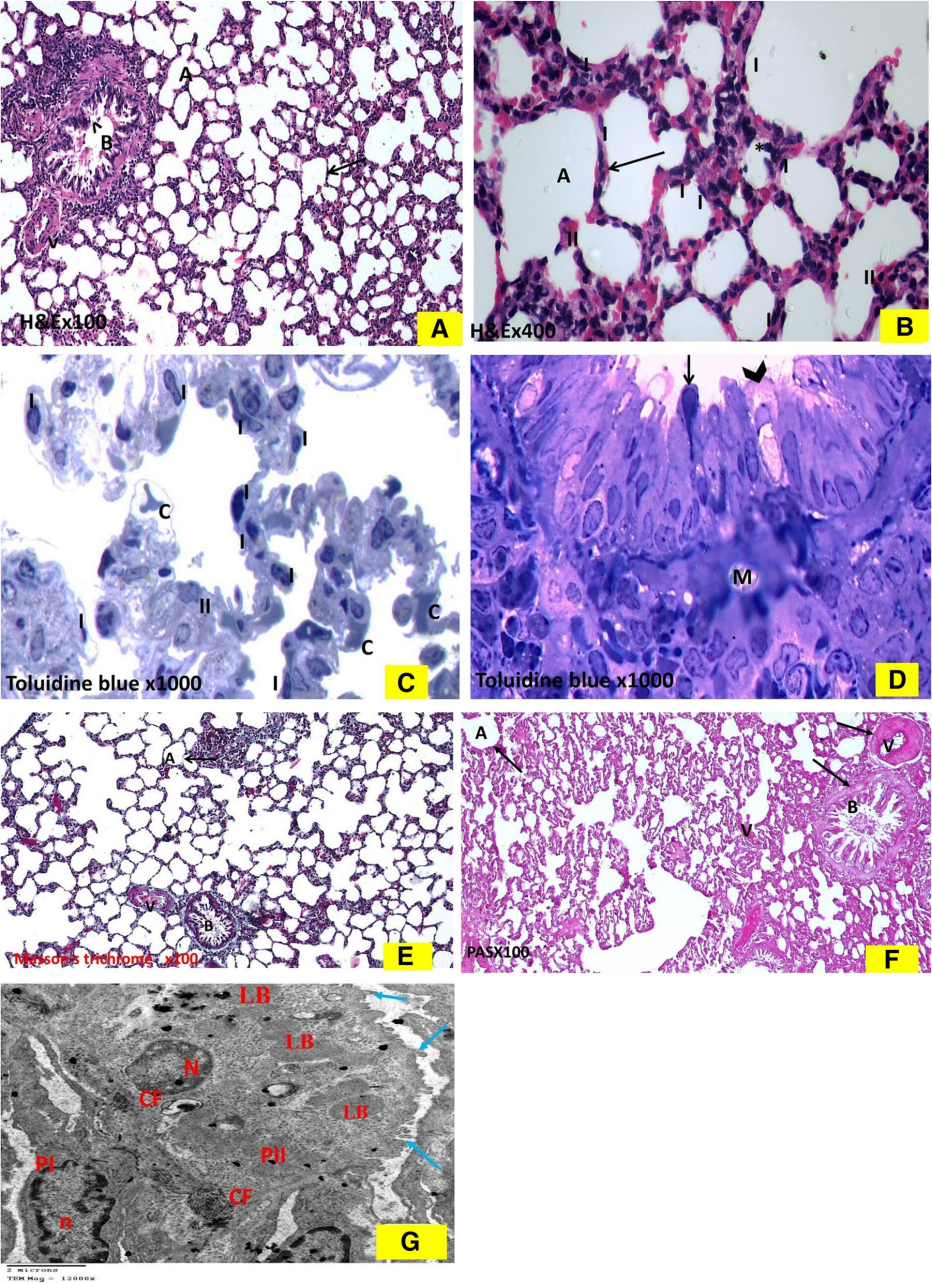
Histological changes in Q- BDL group: The hematoxylin and eosin-stained sections: 6A. Q-BDL group shows relatively thin interalveolar septa (↑) with patent alveoli (A). Bronchiole (B) appears with intact epithelial lining (*^*). Notice a relatively thin walled less congested and less dilated pulmonary blood vessel (V). (× 100). 6 B. Q-BDL shows apparent increase in number of pneumocyte type I (I). Few mononuclear cellular infiltrations (*) are apparent within some relatively thin interalveolar septa (↑), (II) = pneumocyte type II, (A) = alveoli (× 400). Toluidine Blue stained semi thin sections: 6C. Q-BDL group shows relatively thin interalveolar septa with less mononuclear cellular infiltration. Notice the mild congestion of few blood vessels(C) with apparent increase in number of thin type- I pneumocyte with their flat nuclei (I), (II) = pneumocyte type II. (× 1000). 6D. In Q-BDL many of the terminal bronchiole are similar to the control, with its epithelial lining showed two types of cells: ciliated cells (arrow) and non-ciliated cells (Clara cells) (arrowhead). Clara cell has dome shaped apex protruding into the lumen and has narrow base. It had more darkly stained oval indented nucleus, slips of smooth muscle (M) surround the bronchiole also can be seen (× 1000). Photomicrographs of Masson’s trichrome stained sections of rat lung: 6E. The Q-BDL group presents apparent decrease deposition of collagen fibers nearly similar to the sham-operated group in the form of thin fine collagen fibers in the interalveolar septa (↑), per bronchial (B) and perivascular (V) areas, alveoli = (A). (Masson’s trichrome × 100). Photomicrographs of PAS-stained sections of rat lung: 6F. Q-BDL group presents positive PAS reaction (Q-BDL) in bronchiolar passages, wall of a blood vessel, and walls of alveoli. (PAS X 100). 6G. Photomicrographs of Ultrastructure examination by Transmission electron microscope (TEM) of lung: Q-BDL group showing of pneumocyte type-I (PI) with thin attenuated cytoplasm toward the alveolar space and flattened nucleus (n). Dome shaped type-II pneumocyte (PII) and many dense full lamellar bodies (LB) and normal microvilli (red arrow).CF = collagen fibrils (Uranyl acetate and Lead Citrate X120000)

As shown in (Fig. 6), the H & E-stained sections revealed amelioration of the lung injury compared to group II. Most of the lung sections showed restoration of the normal architecture like sham group I in the form of thin interalveolar septa with patent alveolar spaces. Bronchioles appeared with intact epithelial lining. A relatively thin walled, less congested and patent pulmonary blood vessels were noticed (Fig. 6A). Also, apparent increase in the number of pneumocytes type I was noticed. There was limitation of mononuclear cellular infiltration within the relatively thin inter-alveolar septa (Fig. 6B).

The semi thin sections stained by Toluidine blue showed restoration of the normal architecture like sham group I in the form of apparent increase in the number of pneumocytes type I as compared to group II, relatively thin inter-alveolar septa together with less congested capillaries (Fig. 6C). Moreover, semi thin sections stained by Toluidine blue showed many of the terminal bronchioles showed epithelial lining of the two types of cells: ciliated cells and non-ciliated cells (Clara cells) (Fig. 6D).

Masson’s trichrome stained sections of the Q-BDL group presented apparent decrease in the deposition of collagen fibers in the inter-alveolar septa, peri-bronchial and peri-vascular areas nearly similar to the sham-operated group (Fig. 6E). Periodic acid- shiff (PAS) stained sections of the Q-BDL group presented positive PAS reaction in the bronchiolar passages, wall of a blood vessel, and walls of alveoli (Fig. 6F). Transmission electron microscope of the Q-BDL group showed the pneumocyte type-I with thin attenuated cytoplasm toward the alveolar space and flattened nucleus noticed. Together with dome shaped type-II pneumocyte with numerus electron dense full lamellar bodies and normal microvilli (Fig. 6G).

### Immunohistochemical assessment of "e NO"s

**Fig. 7:**
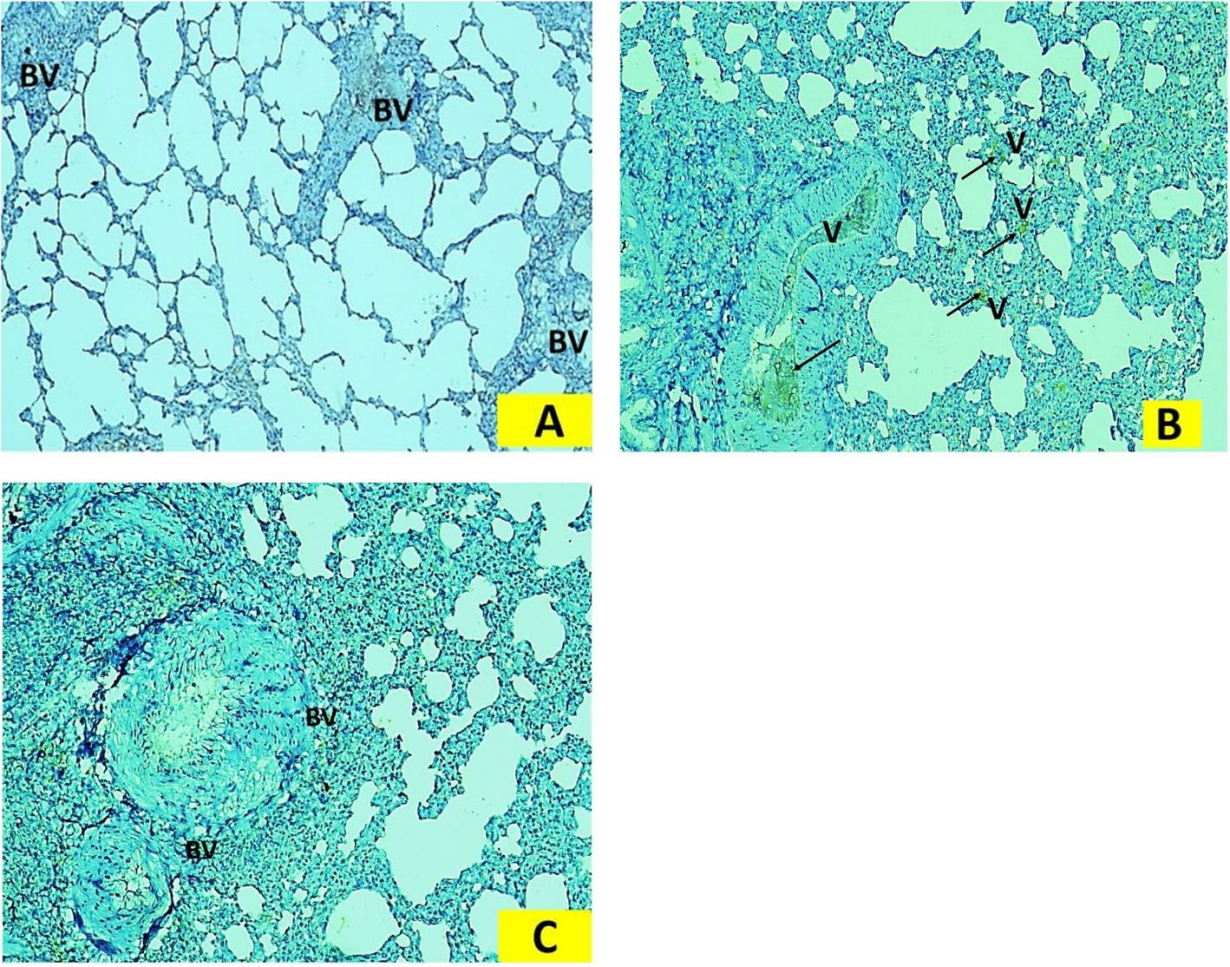
Photomicrographs of "eNO"  s immunohistochemically stained sections of rat lung: 7A. The sham-operated group I showed the week immunostaining of "eNO" s in pulmonary arterial endothelium. Week immune expression seen in the form of absence of brown granules present in the cytoplasm of endothelial cells lining the lumen of the blood vessels (BV). 7B. The BDL group II showed a positive immune reactivity in the form of apparent increase in number of positive immune expression of "eNO"s seen in the pulmonary endothelium (↑), in the form of brown granules present in the cytoplasm of endothelial cells lining the lumen of the blood vessels. 7C. Q-BDL treated group III. There were an apparent reduction of immune reactivity and number of reactive endothelial cells (BV) observed in Q-BDL treated group III compared to the BDL group II. ("eNO" s immunostaining × 400)

As shown in Fig. 7, immunohistochemically stained sections of "eNO"s of the sham-operated group I showed weak immunoexpresion of "e NO"s in pulmonary arterial endothelium (Fig. 7A). In the BDL group positive immune reactivity was seen as brown granules were present in the cytoplasm of endothelial cells lining the lumen of the blood vessels (Fig. 7B). In Q-BDL group apparent reduction of the cytoplasmic immune reactivity and number of reactive endothelial cells of pulmonary blood vessels was observed compared to the BDL group (Fig. 7C).

### Morphometric results (image analysis)

**Fig. 8:**
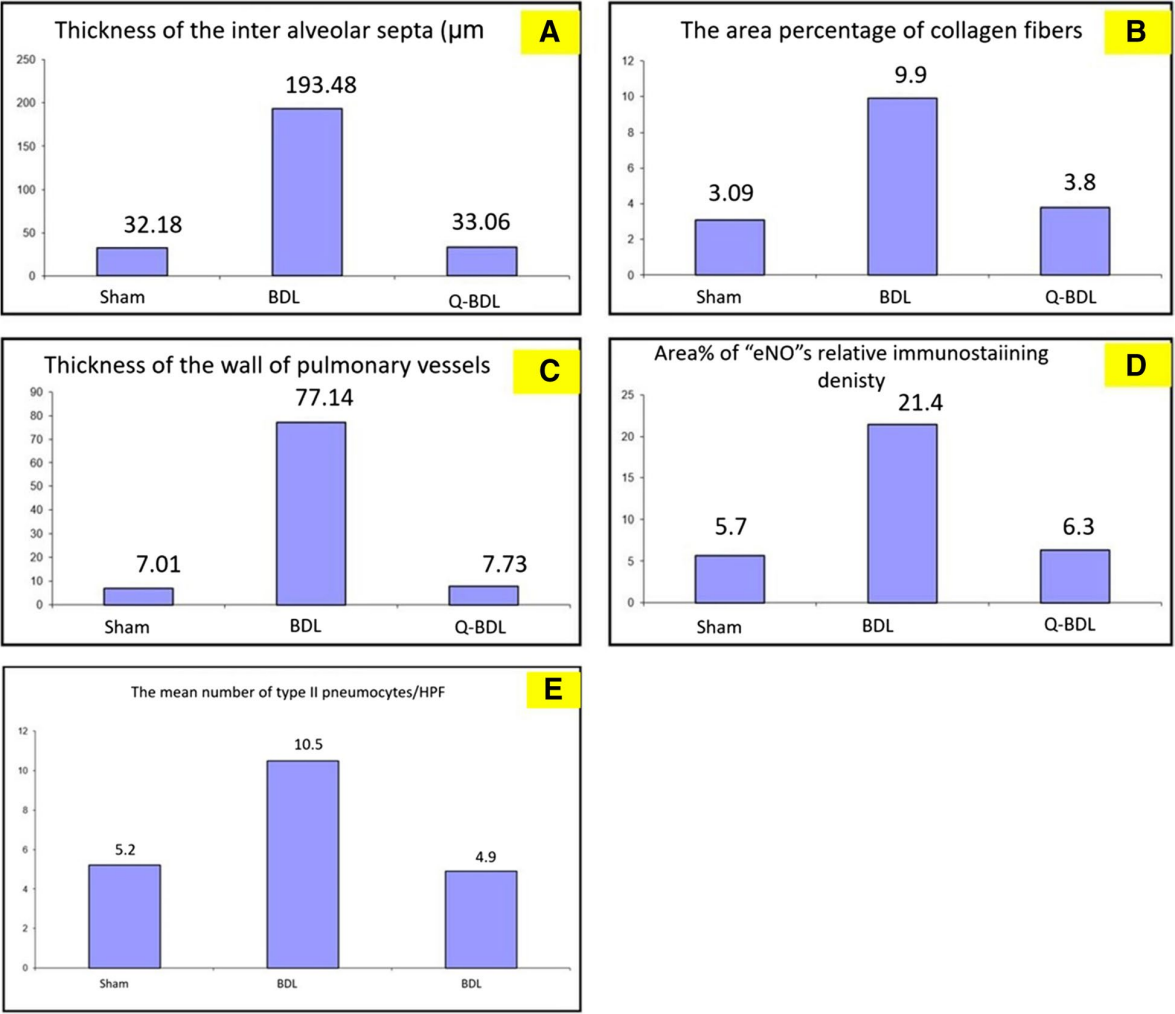
Photomicrographs of morphometric results of lung showing changes per high power field in the three study groups: A. thickness of interalveolar septa. B. thickness of the wall of pulmonary vessels. C. area % for collagen fiber deposition. D. area % for "eNO" s. E. The number of type II pneumocytes/HPF. Data expressed as mean ± SD, and were analysed using one-way ANOVA test. a = Significant at *P* < 0.05 when compared to sham-operated group and calculated by ANOVA, least significant difference. b = Significant at P < 0.05 when compared to BDL group and calculated by ANOVA, least significant difference

There was a significant increase in thickness of interalveolar septa, thickness of the wall of pulmonary vessels and area percentage for collagen fiber deposition in BDL group compared to sham group and Q-BDL. Moreover, there was a significant increase in the number of type II pneumocytes/HPF and area percentage for “eNO” s in BDL group compared to sham group and Q-BDL group. Meanwhile, there was no significance difference between sham group and in Q-BDL group in all these parameters (Tables 4 and 5, Fig. 8A–E).

**Table 4:** Mean ± standard deviation (SD) of the thickness of alveolar septa, thickness of the wall of pulmonary vessels. The mean number of type II pneumocytes /HPF, area percentage of collagen fibers& area % of "eNO" s relative immunostaining density in three experimental groups

Groups	Sham	BDL	Q-BDL
Thickness of the inter alveolar septa (μm)	32.18 ± 0.467	193.48 ± 27.73^a^	33.06 ± 1.9^b^
The area percentage of collagen fibers.	3.09 ± 0.524	9.9 ± 0.803^a^	3.8 ± 0.716^b^
Thickness of the wall of pulmonary vessels	7.01 ± 0.170	77.14 ± 5.318^a^	7.73 ± 0.971^b^
area % of “eNO” s relative immunostaining density	5.7 ± 0.52	21.4 ± 0.61^a^	6.3 ± 0.91^b^
The mean number of type II pneumocytes/HPF	5.2 ± 0.103	10.5 ± 0.562^a^	4.9 ± 0.630^b^

Data expressed as mean ± SD. Data are analyzed using one-way ANOVA and LSD post-hoc test. a = Significant at *P* < 0.05 when compared to sham-operated group calculated by ANOVA, least significant difference. b = Significant at P < 0.05 when compared to BDL group calculated by ANOVA, least significant difference

**Table 5:** Features of acute lung injury to score in H&E stained histological sections of rat lung tissue. The score range is 0 (null) to 8 (severe) for each of the features mentioned

Features	Scoring	Features Description	Sham	BDL	Q-BDL
A – Inflammatory cell		Visible inflammatory cells in air and interstitial spaces	2	8	4
B – Hyaline membranes		Acellular deposit (i.e., devoid of hematoxylin staining) in the alveolar region and stained with eosin	0	8	1
C – Proteinaceous debris		Acellular debris in airspaces	1	7	3
D – Thickening of alveolar wall		Alveolar wall thickening (i.e., at least >1cell layer thick)	1	8	4
E – Enhanced injury		Overall impression of tissue level injury	1	8	3
F – Hemorrhage		Visible red blood cells in the interstitiumor airspaces	1	8	3
G – Atelectasis		complete or partial collapse of distal airspaces	1	6 partial collapse	2
TOTAL SCORE = (sum/56)/ 100			7 12.5%	53 94.6%	20 35.7%

Score0–1; ‘Minimum: absence of injury’, ‘Score 2/3’: mild injury, ‘Score 4’: moderate injury, ‘Score 5/6’: pronounced injury; and ‘Maximum 8’: extensive damage and highest score given

### Semi-quantitative histopathological scoring system for lung injury

As shown in Table 5, In the current work, the sham operated group there was minimum or absence of injury. However, in the BDL group there was extensive damage. Moreover, in the Q-BDL group there was mild to moderate injury. So, Quercetin has potential protective role in acute lung injury induced by bile duct ligation.

## Discussion

Up to the present knowledge, no clear treatments for hepatopulmonary syndrome (HPS), proved to be effective. Providing the safety and ease of quercetin administration, the current work aimed to evaluate the effect of quercetin administration on a rat model of HPS induced by BDL.

Lung indices were significantly increased in the BDL group compared to the control group. This agrees with Bosco et al. [32] who reported increase in pulmonary weight to body weight ratio after common bile duct ligation. This increase was not related to cardiac problems evidenced by the normal cardiac indices in our study.

Histological examination of BDL group showed intrapulmonary angiogenesis, vasodilatation, congestion, thickened interalveolar septa, cellular infiltration and poor recognition for type I and II pneumocyte, together with significant increase in collagen fibers deposition. These findings may explain the elevated lung indices. Lung weight increased because of the congestion and the cellular infiltrate that associated the inflammatory state that developed after bile duct ligation.

In the present work, in BDL group, the inflammatory state, angiogenesis and the intrapulmonary vascular dilatation were postulated to the increased levels of nitric oxide (NO) demonstrated in the area percentage of “eNO” s and ET_B_ receptor expression in lung tissue which were detected by immunohistochemistry and PCR, in the BDL group compared to the sham and Q-BDL groups. The increase in ET-1 activity induces proinflammatory mechanisms, increasing superoxide anion production and cytokine secretion. ET-1 enhances the expression of adhesion molecules on vascular endothelial cells and stimulates the aggregation of polymorphonuclear neutrophils (PMNs) contributing to inflammation and endothelial dysfunction [33]. Moreover, ET-1 act as an angiogenic factor it may upregulate vascular endothelial growth factor and matrix metalloproteins [34]. Further, BDL can trigger recruitment and activation of macrophages in the pulmonary vascular network evidenced by the cellular infiltrate which may produce proinflammatory cytokines, like TNFα leading to NO mediated vasodilatation [2, 35].

Ling et al. [5] and Luo et al. [36] found that hepatic ET-1 derived from proliferating cholangiocytes induces differential effect in the pulmonary circulation depending on this type of receptors (ET-B). The increase detected in endothelin B receptors expression may upregulate “eNO” s and enhance NO production which mediates vascular relaxation. Similar studies reported these effects of BDL in rats, and its ability to produce specific upregulation of the endothelial ET_B_ receptor. Nayyar et al. [37] stated that the increase in pulmonary NO production results from both, increased activity of endothelial nitric oxide synthase (“eNO” s), and inducible nitric oxide synthase (iNOS).

Also, the hyperdynamic circulatory state developed in BDL rats may increase pulmonary blood flow, which goes with Tang et al., [38] and Zopey et al., [39].

Moreover, in agreement with these results Cosarderelioglu et al. [40] and Shikata et al. [41], found that HPS is associated with an increase in the number of pulmonary vessels. The increase in matrix metallopeptidase 9 (MMP-9), TNF-alpha were claimed to cause this angiogenesis. Additionally, Zhang et al. [42] concluded that TNF-alpha produced during inflammation has also been reported to up-regulate vascular endothelial growth factor (VEGF-2) expression and promote angiogenesis and contribute to collateral formation [43].

In the present study, there was statistically significant increase in the thickness of the wall of pulmonary vessels & the thickness of inter alveolar septa leading to gas-exchange abnormalities in BDL group compared to sham and Q-BDL groups. Some of the pulmonary blood vessels showed narrow lumen with apparent intimal damage in the form of endothelial cell disruption, media smooth muscle hypertrophy together with micro thrombi occluding the vessels lumen.

This can be justified by the increased circulating endothelin-1 as it can trigger smooth muscle proliferation, these effects were clarified by Cosarderelioglu et al. [38], DeMartino and Krowka [44] and Raevens and Fallon [6]. Also, the increase in ET-1 may initiate the accumulation of pulmonary intravascular monocytes which produce heme oxygenase (HO-1), that helps in degradation of heme and production of brownish hemosiderin granules that infiltrate the lung tissues in the BDL group [2].

This view was supported by the presence of polymorphic mononuclear inflammatory cells (PMNI) adherent to the disrupted the endothelial cell lining, infiltrating pulmonary blood vessel as well as the interstitium in BDL group.

In the present study, BDL group revealed honeycomb appearance of lungs, as some alveoli are narrowed (atelectasis), while others showed emphysematous dilatation. This was in agreement with Melo-Silva et al. [45] who stated that experimental HPS lead to unequal distribution of alveolar ventilation between the whole alveoli, and decreased mean alveolar length of some alveoli, reflecting alveolar collapse. These functional and structural alveolar alterations in BDL group were associated with signs of degeneration of pneumocyte type I and II, besides the dysfunction in pneumocyte type II cells of some alveoli. Elevated circulating bile acid and TNFα levels, found after BDL might contribute to pneumocyte type II cell dysfunctions [46]. However, the link between cholestasis, alveolar type II cell dysfunction, and HPS has not been clarified yet.

This dysfunction in pneumocyte type II cells was further confirmed by the resultant significant decrease in their surfactant production in BDL group compared to sham and Q -BDL groups. Likewise, Kumer et al. [47] attributed low surfactant production to type II alveolar cells dysfunction. They added that in some types of pulmonary injury, the lung surfactant levels are reduced, and vacuoles are observed in type II cells.

Besides the damage of type II pneumocytes, correlation study in the present work indicates an negative association between endothelin B expression and surfactant concentration in lung tissue as the increase in endothelin B expression in the BDL group was associated with a decrease in the surfactant concentration in lung tissue, giving another explanation for the decrease in surfactant denoting that endothelin may play a role in lung injury. This goes with Comellas and Briva [48] who stated that activation of endothelin B receptors have a role in lung injury, as it recruits inflammatory cells and disrupt endothelial lining.

The significant negative correlation between lung index and surfactant concentration in lung tissues indicates an association between pathological changes in the lung and surfactant concentration in lung tissue. This could be attributed to the loss of the anti-inflammatory effects of surfactant. Surfactant not only prevents collapse, but it also regulates lung injury and inflammation [9]. Jin et al. [49] stated that surfactant protein C dampens inflammation by decreasing JAK/STAT activation.

In this work, Masson’s trichrome stained sections of the BDL group revealed increased collagen fibers deposition in the interalveolar septum, around the bronchioles and perivascular space as described by the morphometric studies Verma [50]. This may be attributed to the up regulation of pro-fibrotic factors from the injured alveolar epithelial cells [51]. Regarding the role of pneumocyte type-II alveolar epithelial cells in lung fibrosis, it was reported that they promote pulmonary fibrosis after acquiring the fibroblast phenotype through epithelial mesenchymal transition [52–54].

Pneumocytes type II appear to be much more responsive to pulmonary irritants than type I cells as it acts as stem cells for regeneration to compensate for the BDL induced alveolar damage and degeneration. Others reported that pneumocytes type II cells are progenitor cells for type I alveolar cells, and after lung injury, they proliferate and restore both types of alveolar cells. Hyperplasia of type II alveolar cells is an important marker of alveolar injury and repair of alveoli [55, 56]. The presence of apparent increase in number, increase in microvilli (blue arrow) reflects signs of proliferation with numerus empty electron lucent lamellar bodies type-II pneumocyte which was similar to what was described by [57] that most type-II pneumocytes were seen with irregular small electron-dense nuclei and contained variable sized empty lamellar bodies in carbon tetrachloride treated rats.

In the present study, the weak expression of PAS staining in the BDL group indicated decreased glycogen content. Reduction in glycogen content may contribute to the clinical manifestations found in HPS as lack of energy, a sensation of exhaustion, fatigue and shortness of breath [58].

In this work, quercetin treated BDL group revealed normal morphology and number of pneumocytes type-II when compared to the BDL group being similar to the sham group. This was associated with restoration of surfactant level to values near sham levels. The results of this work were in agreement with those of Zhang et al. [59] & Li et al. [13].

Also, lung indices decreased being non-significant from the control group. This may be attributed to the ability of quercetin to attenuate inflammation and fibrosis. This partial improvement was evident in the histological picture being similar to the control. As there was statistically significant decrease in the thickness of the inter alveolar septa, thickness of the pulmonary vessels wall and collagen deposition. The morphological and structural improvement were also manifested in the form of apparent fewer pulmonary vascular angiogenesis and less dilated congested pulmonary blood vessels.

Both endothelin 1 beta receptor gene expression and “eNO” s are specific markers for endothelial cells. They were quantitated by PCR and IHC staining analysis respectively. The decrease in both parameters’ levels suggest that quercetin treatment attenuates pulmonary angiogenesis in BDL rats.

Quercetin ability to decrease the inflammation was evidenced by the decrease in inflammatory cell infiltrate, lung indices, endothelin B receptor expression and “eNO” s percentage which will consequently decrease oxidative stress, NF-kappa B activation, and the expression of different pulmonary mediators involved in HPS.

This goes with Tieppo et al. [7] who demonstrated that Quercetin ameliorated liver injury and reduced the expression of hepatic endothelin-1 and HO-1 in cirrhotic rats. These findings suggest that quercetin administered after the onset of hepatic injury significantly may similarly ameliorate pulmonary complications in BDL rats and that also, limitation of cirrhotic evolution may contribute to this effect and improve hypoxemia [60, 61]. Also, different studies stated similar results to this work. Quercetin inhibited the excessive accumulation of extracellular matrix and interstitial fibrosis by antagonizing of NF-kappa B activation [62, 63]. Quercetin administration attenuated lung injury and fibrosis via inhibition of pro-fibrotic cytokines TNF-α and IL-1 [6, 63] denoting its potential anti-fibrotic properties [14, 64]. Aso, Quercetin may inactivate lung macrophage efficiently, decrease the mRNA expression of M1 macrophage markers such as TNF-a, IL-1b, IL-6 and nitric oxide synthase [14, 65, 66].

## Conclusion

Administration of quercetin mitigated lung involvement in experimentally induced hepatopulmonary syndrome by exerting anti-fibrotic, anti-inflammatory, antioxidant, anti-angiogenic effects.

## Data Availability

The data (but not the raw data) of this study are available in this manuscript.

## References

[1] Zhang J, Fallon MB (2012). Hepatopulmonary syndrome: update on pathogenesis and clinical features. Nat Rev Gastroenterol Hepatol.

[2] Soulaidopoulos S, Cholongitas E, Giannakoulas G, Vlachou M, Goulis I (2018). Update on current and emergent data on hepatopulmonary syndromeWorld. J Gastroenterol.

[3] Grilo-Bensusan I, Pascasio-Acevedo J (2016). Hepatopulmonary syndrome: what we know and what we would like to know. World J Gastroenterol.

[4] Gómez F, Barberà J, Roca J, Burgos F, Gistau C, Rodríguez-Roisin R (2006). Effects of nebulized N(G)-nitro-L-arginine methyl ester in patients with hepatopulmonary syndrome. Hepatol.

[5] Ling Y, Zhang J, Luo B, Song D, Liu L, Tang L, Stockard CR, Grizzle WE, Ku DD, Fallon MB (2004). The role of endothelin-1 and the endothelin B receptor in the pathogenesis of hepatopulmonary syndrome in the rat. Hepatol.

[6] Raevens S, Fallon M (2018). Potential clinical targets in hepatopulmonary syndrome: lessons from experimental models. Hepatol.

[7] Tieppo J, Cuevas MJ, Vercelino R, Tuñón MJ, Marroni NP, González-Gallego J (2009). Quercetin administration ameliorates pulmonary complications of cirrhosis in rats. J Nutr.

[8] Gu HJ, Zuo S, Liu HY, Gu LL, Yang XW, Liao J, Wang QQ, Zhao R, Feng XS, Li HY (2019). CX3CR1 participates in pulmonary angiogenesis in experimental hepatopulmonary syndrome mice through inhibiting AKT/ERK signaling pathway and regulating NO/NOS release. Eur Rev Med Pharmacol Sci.

[9] Yang W, Hu B, Wu W, Batra S, Blackburn M, Alcorn J, Fallon M, Zhang J (2014). Alveolar type II epithelial cell dysfunction in rat experimental Hepatopulmonary syndrome (HPS). PLoS One.

[10] Han MK, Barreto TA, Martinez FJ, Comstock AT, Sajjan US (2020). Randomised clinical trial to determine the safety of quercetin supplementation in patients with chronic obstructive pulmonary disease. BMJ Open Respir Res.

[11] Lu J, Wang Z, Li S, Xin Q, Yuan M, Li H, Song X, Gao H, Pervaiz N, Sun X, Lv W (2018). Quercetin inhibits the migration and invasion of HCCLM3 cells by suppressing the expression of p-Akt1, matrix metalloproteinase (MMP) MMP-2, and MMP-9. Med Sci Monit.

[12] Li X, Chen Y, Wang L, Shang G, Zhang C, Zhao Z, Zhang H, Liu A (2016). Quercetin alleviates pulmonary angiogenesis in a rat model of hepatopulmonary syndrome. Braz J Med Biol Res.

[13] Li X, Jin Q, Yao Q, Xu B, Li L, Zhang S, Tu C (2018). The flavonoid quercetin ameliorates liver inflammation and fibrosis by regulating hepatic macrophages activation and polarization in mice. Front Pharmacol.

[14] Araújo NPDS, de Matos NA, Oliveira M, de Souza ABF, Castro TF, Machado-Júnior PA, de Souza DMS, Talvani A, Cangussú SD, de Menezes RCA, Bezerra FS (2022). Quercetin improves pulmonary function and prevents emphysema caused by exposure to cigarette smoke in male mice. Antioxidants (Basel).

[15] National Institutes of Health (U.S). Institutional Animal Care and Use cmmittee guidebook. second edition. published by, Office of Laboratory Animal Welfare, National Institutes of Health; 2002.

[16] Declaration of Helsinki 1964. Recommendations guiding physicians in biomedical research involving human subjects. BMJ. 1996;313:1448.

[17] Abushady EA, Elagaty SM, Nassef NA, Abdelhamid GS. The potential hepatoprotective effect of quercetin on cholestatic liver injury in rats. Bull Egypt Soc Physiol Sci. 2019;40(1):84–95.

[18] Tag CG, Sauer-Lehnen S, Weiskirchen S, Borkham-Kamphorst E, Tolba RH, Tacke F, and Weiskirchen F. Bile duct ligation in mice: induction of inflammatory liver injury and fibrosis by obstructive cholestasis. J Vis Exp. 2015;10(96). 10.3791/52438 PMC4354634 25741630

[19] Zhao Y, Zhang M, Xiong RP, Chen XY, Li P, Ning YL, Yang N, Peng Y, Zhou YG (2016). Somatostatin reduces the acute lung injury of mice via increasing the affinity of glucocorticoid receptor. Cell Physiol Biochem.

[20] Balyakina EV, Chen D, Lawrence ML, Manning S, Parker RE, Shappell SB, Meyrick B (2002). ET-1 receptor gene expression and distribution in L1 and L2 cells from hypertensive sheep pulmonary artery. Am J Physiol Lung Cell Mol Physiol..

[21] Black JG, Black LJ (2018). Microbiology: principles and explorations.

[22] Widbiller M, Rothmaier C, Saliter D, Wölflick M, Rosendahl A, Buchalla W, Schmalz G, Spruss T, Galler KM (2021). Histology of human teeth: standard and specific staining methods revisited. Arch Oral Biol.

[23] Lu JW, Yang WY, Lin YM, Jin SL, Yuh CH (2013). Hepatitis B virus X antigen and aflatoxin B1 synergistically cause hepatitis, steatosis and liver hyperplasia in transgenic zebrafish. Acta Histochem.

[24] Drury R, Carleton WE (1980). Histological techniques.

[25] Bancroft JD, Gamble M (2002). Theory and Practice. In: Histological Techniques.

[26] Sridharan G, Shankar AA (2012). Toluidine blue: a review of its chemistry and clinical utility. J Oral Maxillofac Pathol.

[27] Suvarna K, Layton C, Bancroft J. Theory and practice of histological techniques, vol. 203. 7th ed. Churchill Livingstone, USA; 2013. p. 500.

[28] Weidenheim KM, Hinchey WW, Campbell WG (1983). Hyperinsulinemic hypoglycemia in adults with islet-cell hyperplasia and degranulation of exocrine cells of the pancreas. Am J Clin Pathol.

[29] Marković Filipović J, Miler M, Kojić D, Karan J, Ivelja I, Čukuranović Kokoris J, Matavulj M. Effect of acrylamide treatment on Cyp2e1 expression and redox status in rat hepatocytes. Int J Mol Sci. 2022;23(11):6062. 10.3390/ijms23116062 PMC9181519 35682741

[30] Silva AI, Gvazava N, Bölükbas D, Stenlo M, Dong J, Pierre L, et al. A semi-quantitative scoring system for green histopathological evaluation of large animal models of acute lung injury. Bio-protocol. 2022;12(16). 10.21769/BioProtoc.4493 PMC9486691 36199700

[31] Sawilowsky S (2005). Misconceptions leading to choosing the t test over the Wilcoxon Mann-Whitney U test for shift in location parameter. J Mod Appl Stat Methods.

[32] Bosco AD, Schedler FB, Colares JR, Schemitt EG, Hartmann RM, Forgiarini Junior LA, Dias AS and Marroni NP. Melatonin effects on pulmonary tissue in the experimental model of Hepatopulmonary syndrome. J Bras Pneumol. 2019;45(3):e20170164. 10.1590/1806-3713/e20170164. 10.1590/1806-3713/e20170164 PMC6715043 31166552

[33] Kowalczyk A, Kleniewska P, Kolodziejczyk M, Skibska B, Goraca A. The role of endothelin-1 and endothelin receptor antagonists in inflammatory response and sepsis. Arch Immunol Ther Exp. 2015;63(1):41–52. 10.1007/s00005-014-0310-1. 10.1007/s00005-014-0310-1 PMC4289534 25288367

[34] Wu MH, Huang CY, Lin JA, Wang SW, Peng CY, Cheng HC, Tang CH (2014). Endothelin-1 promotes vascular endothelial growth factor-dependent angiogenesis in human chondrosarcoma cells. Oncogene.

[35] Zhang HY, Han DW, Su AR, Zhang LT, Zhao ZF, Ji JQ, Li BH, Ji C (2007). Intestinal endotoxemia plays a central role in development of hepatopulmonary syndrome in a cirrhotic rat model induced by multiple pathogenic factors. World J Gastroenterol.

[36] Luo B, Tang L, Wang Z, Zhang J, Ling Y, Feng W, Sun J, Stockard C, Frost A, Chen Y. Cholangiocyte Endothelin 1 and Transforming Growth Factor β1 Production in Rat Experimental Hepatopulmonary Syndrome. Gastroenterology. 2005;129(2):682–95. 10.1016/j.gastro.2005.05.050 PMC2830806 16083721

[37] Nayyar D, Man HS, Granton J, Lilly LB, Gupta S (2015). Proposed management algorithm for severe hypoxemia after liver transplantation in the hepatopulmonary syndrome. Am J Transplant.

[38] Tang L, Luo B, Patel RP, Ling Y, Zhang J, Fallon MB (2007). Modulation of pulmonary endothelial endothelin B receptor expression and signaling: implications for experimental hepatopulmonary syndrome. Am J Physiol Lung Cell Mol Physiol.

[39] Zopey R, Susanto I, Barjaktarevic I, Wang T. Transition from hepatopulmonary , syndrome to portopulmonary hypertension: A case series of 3 patients. Case Rep Pulmonol. 2013;561870. 10.1155/2013/561870 PMC3844212 24324910

[40] Cosarderelioglu C, Cosar AM, Gurakar M, Dagher NN, Gurakar A (2016). Hepatopulmonary syndrome and liver transplantation: a recent review of the literature. J Clin Transl Hepatol.

[41] Shikata F, Sakaue T, Nakashiro K-i, Okazaki M, Kurata M, Okamura T, Okura M, Ryugo M, Nakamura Y, Yasugi T, Higashiyama S,Izutani H. Pathophysiology of lung injury induced by common bile duct ligation in mice. PLoS One. 2014;9(4):e94550. 10.1371/journal.pone.0094550 PMC3986091 24733017

[42] Zhang J, Ling Y, Tang L, Luo B, Pollock DM, Fallon MB (2009). Attenuation of experimental hepatopulmonary syndrome in endothelin B receptor-deficient rats. Am J Physiol Gastrointest liver Physiol.

[43] Thenappan T, Goel A, Marsboom G, Fang YH, Toth PT, Zhang HJ, Kajimoto H, Hong Z, Paul J, Wietholt C, Pogoriler J (2011). A central role for CD68+ macrophages in hepatopulmonary syndrome. Reversal by macrophage depletion. Am J Respir Crit Care Med.

[44] DeMartino ES, Krowka MJ (2017). Pulmonary vascular complications of liver disease. Int Anesthesiol Clin.

[45] Melo-Silva CA, Gaio E, Trevizoli JE, Souza CS, Goncalves AS, Sousa GC, Takano G, Tavares P, Amado VM (2011). Respiratory mechanics and lung tissue remodeling in a hepatopulmonary syndrome rat model. Respir Physiol Neurobiol.

[46] Horvatits T, Drolz A, Rutter K, Roedl K, Fauler G, Muller C, Kluge S, Trauner M, Schenk P, Fuhrmann V (2017). Serum bile acids in patients with hepatopulmonary syndrome. Z Gastroenterol.

[47] Kumar V, Abbas AK, Aster JC (2017). Robbins basic pathology e-book.

[48] Comellas AP, Briva A, Dada LA, Butti ML, Trejo HE, Yshii C, Azzam ZS, Litvan J, Chen J, Lecuona E, Pesce LM, Yanagisawa M, Sznajder JI (2009). Endothelin-1 impairs alveolar epithelial function via endothelial ETB receptor. Am J Respir Crit Care Med.

[49] Jin H, Ciechanowicz AK, Kaplan AR, Wang L, Zhang PX, Lu YC, Tobin RE, Tobin BA, Cohn L, Zeiss CJ, Lee PJ, Bruscia EM, Krause DS (2018). Surfactant protein C dampens inflammation by decreasing JAK/STAT activation during lung repair. Am J Physiol Lung Cell Mol Physiol.

[50] Verma R, Kushwah L, Gohel D, Patel M, Marvania T, Balakrishnan S (2013). Evaluating the ameliorative potential of quercetin against the bleomycin-induced pulmonary fibrosis in Wistar rats. Pulm Med.

[51] Elliott MR, Koster KM, Murphy PS (2017). Efferocytosis signaling in the regulation of macrophage inflammatory responses. J Immunol.

[52] Zhang E, Yang Y, Chen S, Peng C, Lavinm MF, Yeo AJ, Li C, Liu X, Guan Y, Du X, Du Z (2018). Bone marrow mesenchymal stromal cells attenuate silica-induced pulmonary fibrosis potentially by attenuating Wnt/β-catenin signaling in rats. Stem Cell Res Ther.

[53] Kim KK, Dotson MR, Agarwal M, Yang J, Bradley P, Subbotina N, Osterholzer JJ, Sisson TH (2018). Efferocytosis of apoptotic alveolar epithelial cells is sufficient to initiate lung fibrosis. Cell Death Dis.

[54] Guoa J, Yangb Z, Jiab Q, Bob C, Shaob H, Zhang Z (2019). Pirfenidone inhibits epithelial-mesenchymal transition and pulmonary fibrosis in the rat silicosis model. Toxicol Lett.

[55] Serrano-Mollar A (2018). Cell therapy in idiopathic pulmonary fibrosis. Med Sci (Basel).

[56] Guillamat-Prats R, Camprubí-Rimblas M, Puig F, Herrero R, Tantinyà N, Serrano-Mollar A. Artigas a (2020): alveolar type II cells or mesenchymal stem cells: comparison of two different cell therapies for the treatment of acute lung injury in rats. Cells. 1816;9(8). 10.3390/cells9081816 PMC7464506 32751857

[57] Hamam MHKK, Raafat MH (2019). Histological study on possible therapeutic effect of BM-MSCs on healing of lung fibrosis induced by CCl4 with reference to macrophage plasticity. J Cytol Histol.

[58] Borges EL, de Barros PM, Prata LO, Sales WA, Silva YA, Caliari MV, Roderigues-MachadoMG. Effect of lung fibrosis on glycogen content in different extrapulmonary tissues. Lung. 2014;192(1):125–31. 10.1007/s00408-013-9539-4 24297324

[59] Zhang J, Luo B, Tang L, Wang Y, Stockard CR, Kadish I, Van Groen T, Grizzle WE, Ponnazhagan S, Fallon MB (2009). Pulmonary angiogenesis in a rat model of hepatopulmonary syndrome. Gastroenterol.

[60] Yang Y, Chen B, Chen Y, ZuB YB, Lu K (2015). A comparison of two common bile duct ligation methods to establish hepatopulmonary syndrome animal models. Lab Anim.

[61] Liu X, Zhang Y, Liu L, Pan Y, Hu Y, Yang P, Liao M (2020). Protective and therapeutic effects of nanoliposomal quercetin on acute liver injury in rats. BMC Pharmacol Toxicol.

[62] Elbe H, Dogan Z, Taslidere E, Cetin A, Turkoz Y (2016). Beneficial effects of quercetin on renal injury and oxidative stress caused by ciprofloxacin in rats: a histological and biochemical study. Hum Exp Toxicol.

[63] Diniz LRL, Souza MTS, Duarte ABS (2020). Sousa DP (2020): mechanistic aspects and therapeutic potential of quercetin against COVID-19-associated acute kidney injury. Mole.

[64] Zhang K, Lin S, Wang M, Huang J, Zhu Y (2020). The Risk of Acute Kidney Injury in Hepatitis B Virus-Related Acute on Chronic Liver Failure with Tenofovir Treatment. Biomed Res Int.

[65] Lu H, Wu L, Liu L, Ruan Q, Zhang X, Hong W, Wu S, Jin G, Bai Y (2018). Quercetin ameliorates kidney injury and fibrosis by modulating M1/M2 macrophage polarization. Biochem Pharmacol.

[66] Tan RZ, Wang C, Deng C, Zhong X, Yan Y, Luo Y, Lan HY, He T, Wang L (2020). Quercetin protects against cisplatin-induced acute kidney injury by inhibiting Mincle/Syk/NF-κB signaling maintained macrophage inflammation. Phytother Res.

